# Supplementary figures and images for: Common, low-frequency, rare, and ultra-rare coding variants contribute to COVID-19 severity
Source: Hum Genet. 2021 Dec 10;141(1):147–73. doi: 10.1007/s00439-021-02397-7 (PMC8661833; doi:10.1007/s00439-021-02397-7)

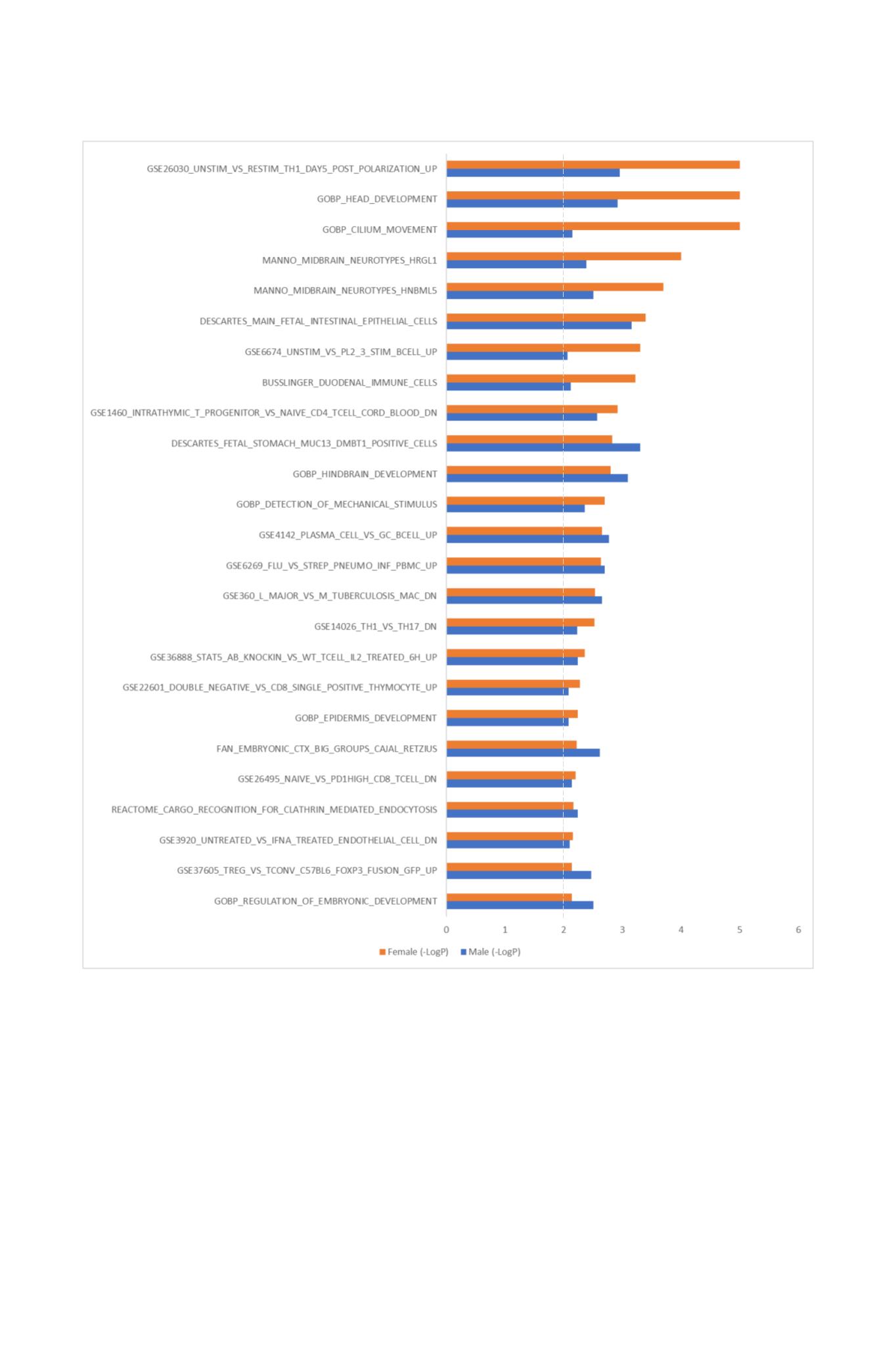

Supplement: Supplementary file 1 — Supplementary Figure 1. Barplots for 0.01 both. Barplot of significance values (NOM p-values, -log10 transformation) from GSEA analysis for all the pathways significant in both females (orange) and males (blue), p<0.01. Vertical dotted line indicates the adopted significance threshold (JPG 130 kb) [file 439_2021_2397_MOESM1_ESM.jpg]

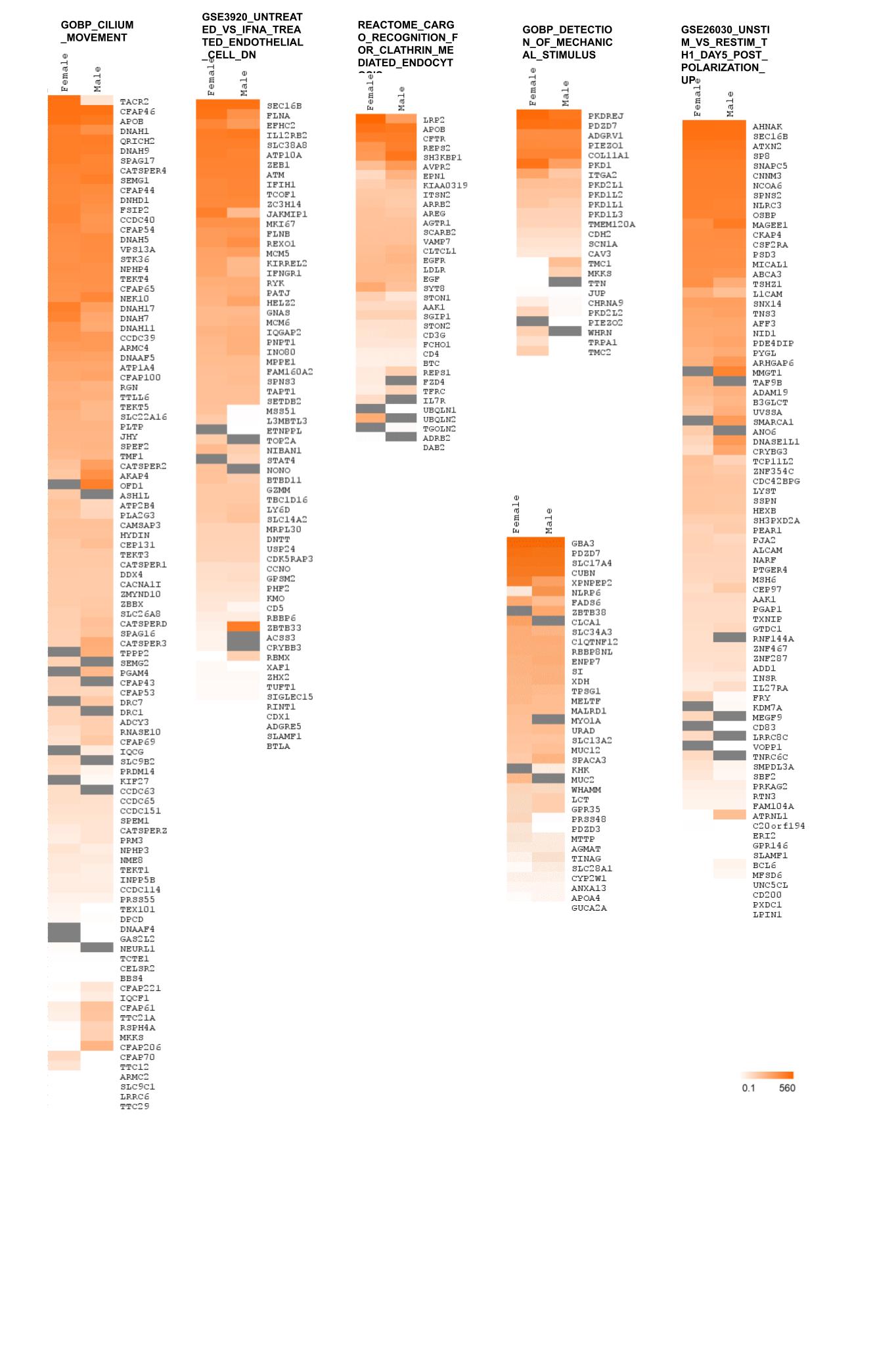

Supplement: Supplementary file 2 — Supplementary Figure 2. Representative heatmaps for 0.01_both. Heatmaps of the genes belonging to representative pathways significant in both females and males, p<0.01. The color gradient represents the weight of each gene, calculated as described in methods (JPG 210 kb) [file 439_2021_2397_MOESM2_ESM.jpg]

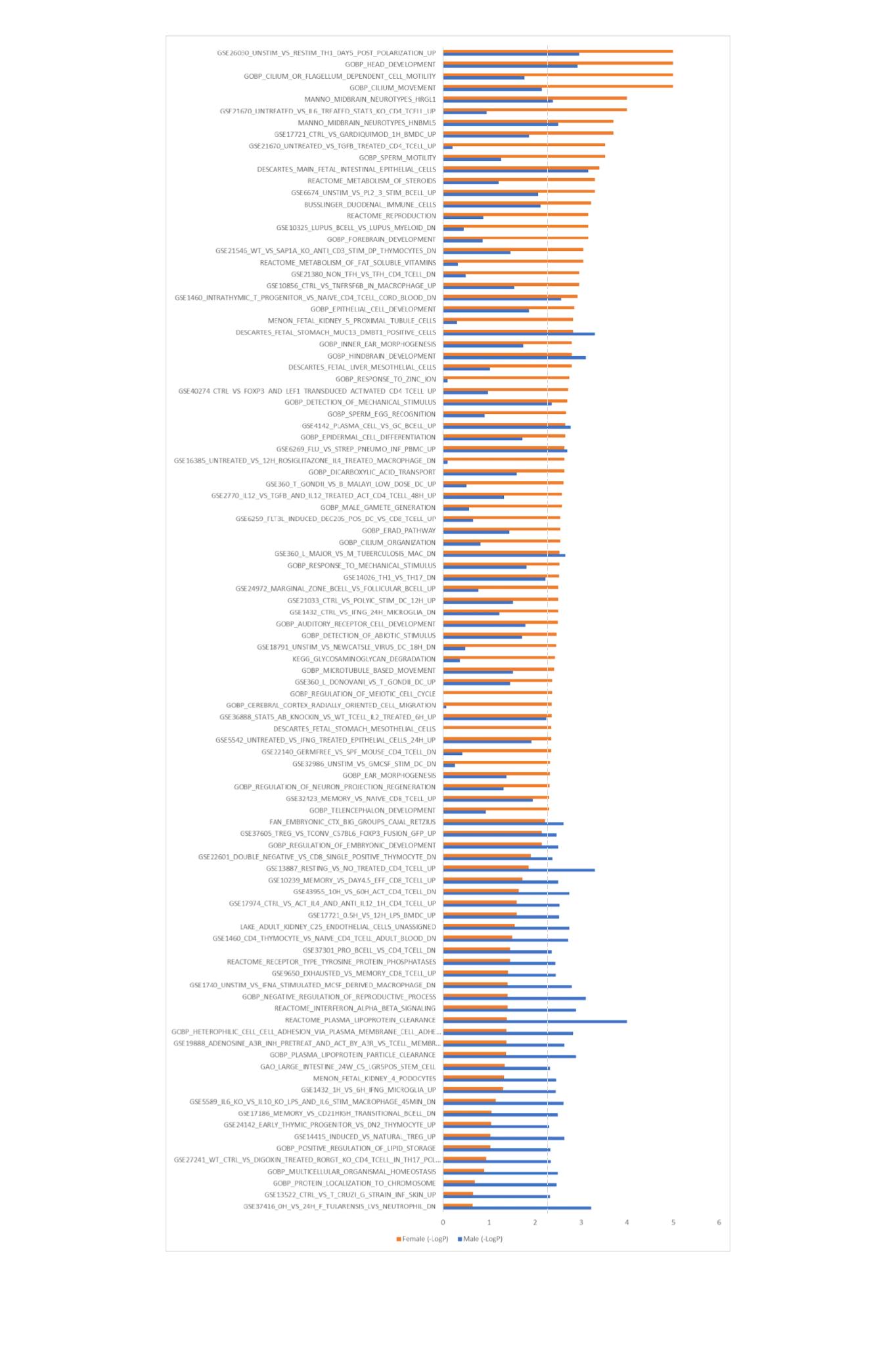

Supplement: Supplementary file 3 — Supplementary Figure 3. Barplots for 0.005_any. Barplot of significance values (NOM p values, -log10 transformation) from GSEA analysis for all the pathways significant in either females (orange) or males (blue), p<0.005. Vertical dotted line indicates the adopted significance threshold (JPG 237 kb) [file 439_2021_2397_MOESM3_ESM.jpg]

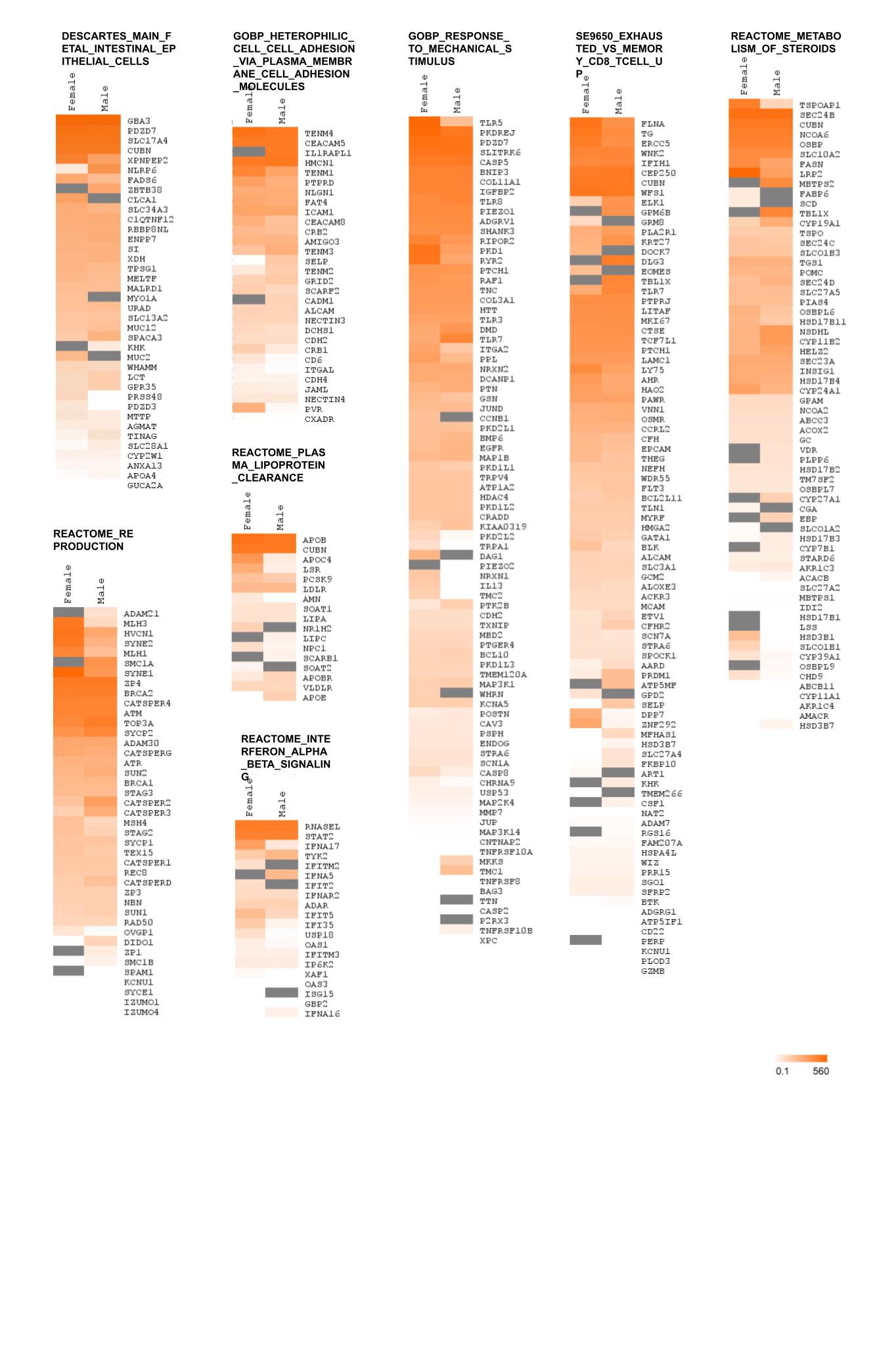

Supplement: Supplementary file 4 — Supplementary Figure 4. Representative heatmaps for 0.005_any. Heatmaps of the genes belonging to representative pathways significant in either females or males, p<0.005. The color gradient represents the weight of each gene, calculated as described in methods (JPG 223 kb) [file 439_2021_2397_MOESM4_ESM.jpg]
